# Green authenticity and resident subjective wellbeing: large-scale sport events as social interventions for public health

**DOI:** 10.3389/fpubh.2026.1876921

**Published:** 2026-07-02

**Authors:** Jiexin Chen, Yao Zhang, Ji Wu

**Affiliations:** 1 School of Economics and Management, Shanghai University of Sport, Shanghai, China; 2 School of Marxism, South China University of Technology, Guangzhou, China

**Keywords:** green initiatives, host communities, service-dominant logic, subjective wellbeing, value co-creation

## Abstract

**Background:**

Subjective wellbeing is a core public health priority. Previous research overlooked the impact of large-scale sport events on improving subjective wellbeing among host residents. Grounded in Service-Dominant Logic, this study investigated how residents’ perceptions of green initiative authenticity and green value congruence influence subjective wellbeing.

**Methods:**

Using a cross-sectional survey, 823 valid responses were collected during the 2023 Hangzhou Asian Games, with data analyzed via Partial Least Square Structural Equation Modeling.

**Results:**

Results show that residents’ perceptions of green initiative authenticity and green value congruence positively predicted value co-creation, which in turn enhanced subjective wellbeing. Value co-creation fully mediated the relationship between perceived authenticity and subjective wellbeing, while it partially mediated the association between value congruence and subjective wellbeing.

**Conclusion:**

This study extends Service-Dominant Logic application to passive residents, enriches sport event Social Leverage Theory, and provides actionable insights for organizers to design authentic, community-aligned green initiatives. Therefore, large-scale sport events as viable public health interventions can enhance sustainability and community wellbeing by fostering inclusive value co-creation.

## 1 Introduction

Subjective wellbeing (SWB), defined as individuals’ cognitive evaluations of life satisfaction and experiences of positive emotions (1, 2), has become a central concern in public health globally. The World Health Organization emphasizes that SWB serves not only as a personal outcome but also as a collective marker of community resilience, social cohesion, and equitable development. In contemporary cities, however, urbanization, social disconnection, and environmental decline continue to erode wellbeing, contributing to heightened stress and widening health disparities (3, 4). Addressing these issues calls for innovative, community-oriented interventions that look beyond clinical settings and draw on the resources already embedded in communities themselves.

One increasingly recognized strategy is social prescribing. It focuses on a person-centered approach that connects people with non-clinical activities and supports in their local environments, such as nature engagement, arts programs, and sport-based initiatives. Recent research has increasingly highlighted large-scale sports events, such as the Olympic Games, the FIFA World Cup, and Asian Games, as promising platforms for community-based health promotion (2, 5, 6). Beyond their economic and cultural impact, these events create unique opportunities to engage communities, improve local environments, and strengthen social bonds, all of which are established determinants of SWB (2, 7, 8). Indeed, enhancing the SWB of the local residents has become a central rationale for cities bidding to host large-scale sport events (9, 10). However, the existing literature on sports events and SWB has two critical gaps. First, most studies have focused on active event stakeholders, including spectators, athletes, and volunteers, while neglecting the host community residents who engage with events passively. This oversight undermines the equity of public health gains, as meaningful wellbeing improvements must extend beyond a small subset of participants to benefit the entire community. Second, existing research has predominantly explained the SWB effects of sports through a “feel good effect” without fully elucidating the underlying pathways. According to Chalip (11), simply hosting a sporting event does not guarantee positive outcomes. Instead, benefits are achieved by strategically integrating peripheral elements and initiatives (12). Although environmental sustainability has become a central pillar of modern sports events, few studies have examined how residents’ perceptions of such green initiatives translate into improved wellbeing outcomes.

To address these gaps, the present study draws on Service-Dominant Logic (SDL) (13), a framework that reframes value creation as a collaborative process involving multiple stakeholders, such as event organizers, residents, and communities, through resource integration (14–16). From an SDL standpoint, large-scale sport events function as dynamic platforms where residents are not passive recipients of benefits but active resource integrators, contributing their time, knowledge, and behaviors to create shared value (15, 17, 18). In the context of environmental sustainability, this implies that green initiatives enhance public health outcomes only when residents perceive them as authentic and aligned with their community needs. These perceptions, in turn, motivate residents to engage in green value co-creation, such as volunteering for waste sorting or adopting low-carbon behaviors, which ultimately fulfills the psychological needs that underpin SWB. However, this mechanism has not yet been examined empirically.

The purpose of the current study was to investigate two core research questions: (1) How do residents perceive the green initiatives of large-scale sporting events? (2) How do these perceptions predict residents’ subjective wellbeing? These questions were addressed through an empirical investigation of the 2023 Hangzhou Asian game (2023HAG). The 2023HAG was selected as a research context for being the first Asian Games to fully integrate United Nations Sustainable Development Goals into its planning.

This study makes two key contributions to the sports and public health literature. First, it positions large-scale sports events as viable public health interventions by demonstrating how event-green initiatives can be leveraged to enhance the SWB of residents who are passively engaged in large-scale sporting events. Second, it operationalizes SDL to elucidate the mechanisms connecting event initiatives to public health outcomes, highlighting the critical role of authenticity, congruence, and resource integration in driving meaningful wellbeing gains. These findings provide initial insights for public health practitioners, event organizers, and policymakers seeking to design inclusive and sustainable events that may promote residents’ health and wellbeing.

## 2 Literature review

### 2.1 Subjective wellbeing

SWB refers to an individual’s subjective evaluation of their life, encompassing both cognitive judgments and affective experiences (1). Specifically, the cognitive component pertains to how individuals assess the influence of life events on their SWB, whereas the affective component involves the evaluation of mood and emotions arising from those events (1, 19). Expanding on this, Diener et al. (20) emphasized that “people have wellbeing only when they believe that their life is going well’ (p. 195).

Prior research has documented increases in SWB among active participants, such as visitors, spectators, and players, during large-scale sport events (2, 8, 9). Nevertheless, findings on the event-specific factors that influence SWB have yielded inconclusive results. Drawing on Social Leverage Model (11), many studies have shown that event attributes such as entertainment, celebration, and social interactions can enhance individuals’ SWB (2, 7, 9). This body of research contends that active participation in sports events fosters SWB by cultivating feelings of pride, reinforcing a sense of belonging, and offering escape from daily stress (7, 9). In contrast, Asan et al. (21) observed no significant effect of entertainment experiences on individuals’ SWB during events; instead, they found that residents’ SWB was positively influenced by their perceptions of community-level development driven by sports events.

A review of the literature revealed that most studies on SWB in the context of large-scale sports events have focused on active participant groups (2, 9, 22, 23). In comparison, research focusing specifically on host community residents remains relatively scarce despite their status as a critical stakeholder group whose perceptions and wellbeing significantly shape an event’s sustainability and developmental outcomes. Accordingly, the question of how event hosting influences residents’ SWB warrants further investigation. Notably, emerging evidence suggests that sports events can enhance SWB not only among active participants but also among residents in host communities (2, 19, 24). For instance, Zhao et al. (24) demonstrated that the event environment, such as ambient conditions and symbolic elements, strengthens the place attachment of residents and encourages value co-creation behaviors, thereby elevating their SWB. The current study examined whether host community residents perceive green initiatives associated with large-scale sports events as authentic and congruent with local values and how such perceptions influence their SWB. Aligned with Diener et al.’s (20) conceptualization, we operationalized SWB as a self-reported assessment of quality of life and emotional states, reflecting internally held personal standards.

### 2.2 Service dominant logic

SDL represents a transformative paradigm that redefines value creation as a process centered on service exchange, rather than the mere distribution of goods (13). Originating in marketing, this framework posits that value is not embedded in outputs, but is co-created through the integration of diverse resources among multiple actors. Beneficiaries ultimately determine value based on their unique experiences and resource integration processes (13, 25). The core mechanism of SDL operates through reciprocal service exchange. From this perspective, entities such as event organizers do not deliver finished values, but instead provide value propositions. These propositions invite actors, including customers, sponsors, and community residents, to share and integrate resources such as private, market-facing, and public resources (16, 26, 27). The effective integration of these resources gives rise to value co-creation. Owing to its relevance in explaining dynamic value formation, SDL has been broadly applied across disciplines, such as marketing, tourism, and event management (15, 17, 26). Grounded in this framework, this study focuses on how large-scale sports events as platforms for service exchange improve residents’ SWB.

### 2.3 Sport event as a space for value co-creation

From the lens of SDL, sport events function as dynamic arenas for value co-creation, bringing together spectators, residents, athletes, and organizers within the pre-planned temporal and physical domains. Within these spaces, diverse stakeholders integrate operant, such as knowledge and culture, with operand resources, such as facilities and labor, to generate mutual value (14, 15, 28). Such an environment enables stakeholders to contribute unique resources, collectively shape the meaning of sports events, and derive personal and communal benefits.

Prior research has demonstrated that residents’ engagement in value co-creation at sporting events is shaped by their perceptions of event impact, tourism development, and liminal experiences (11, 18, 19). These perceptions have been shown to enhance SWB (2, 7, 16, 17). Existing studies suggest that the exchange of social bonds, the experience of positive emotions, and tangible improvements in living conditions and infrastructure collectively create value that manifests as improved SWB. For instance, Horbel et al. (16) found that residents, acting as resource integrators, enhance their SWB through event-related social interactions with visitors and by accessing legacy resources. Similarly, Zhao et al. (24) identified enhanced living conditions as the most influential driver of SWB, following Chengdu University in 2021. To further refine this relationship, Chen et al. (19) and Asan et al. (21) proposed that the value co-creation process, along with educational and escapist experiences, serves as a key mediator between event engagement and SWB within sports settings.

This study centers on residents’ value co-creation intention, the initial stage of the co-creation process. As widely acknowledged in behavioral research, intention serves as the immediate antecedent of actual behaviors (13, 17, 24). In this study, we primarily focus on the formation mechanism of residents’ green co-creation intention within the large-scale sport event. From the SDL perspective, residents’ willingness to share environmental knowledge and participate in green initiatives represents a readiness for resource integration (17). When supported by favorable event atmosphere, social norms and convenient participation conditions, such psychological readiness will be converted into observable health and environmental behaviors.

### 2.4 Authenticity

Authenticity is defined as the quality of genuine, original, or consistent with an entity’s inherent values. It is commonly conceptualized through three core dimensions: (1) objective authenticity, which concerns the historical or material integrity of artifacts or cultural relics (29, 30); (2) existential authenticity, which represents subjective feelings of self-realization and personal meaning derived from experiences (31, 32); and (3) constructive authenticity, which reflects the subjective interpretation formed through sensory engagement, situational interactions, and personal evaluations (30). This study focused on residents’ perceptions of the authenticity of event-related green initiatives. Accordingly, we adopt a constructive authenticity perspective that aligns with the interpretive and socially constructed nature of such perceptions. Hence, we defined the perceived authenticity of green event initiatives as residents’ subjective evaluation of whether these pro-environmental practices are genuine, aligned with core sustainability values, and responsive to community needs (30, 31).

From an objective standpoint, the perceived authenticity of green event initiatives depends on tangible environmental legacies. As noted by Chen et al. (19), residents assess authenticity based on the substantive implementation of initiatives, such as the use of solar-powered stadiums and carbon-neutral operations during the 2023HAG, and their long-term environmental impacts, including the preservation of green spaces after the event (29). Such evaluations tend to favor meaningful actions over superficial symbolic measures. Subjectively, perceived authenticity hinges on the alignment between green initiatives and a community’s sustainability agenda and cultural contexts (27, 29, 31). Residents are more likely to perceive high authenticity when event green initiatives reflect local ecological priorities and avoid perceptions of “greenwashing,” a concern also highlighted in authenticity research (32, 33). When authenticity is perceived, it fosters trust in event organizers and encourages sustained pro-environmental actions among residents after the event (30, 33), underscoring its dual character as a quality grounded in objective reality yet shaped through social interpretation.

## 3 Hypotheses development

**Figure 1:**
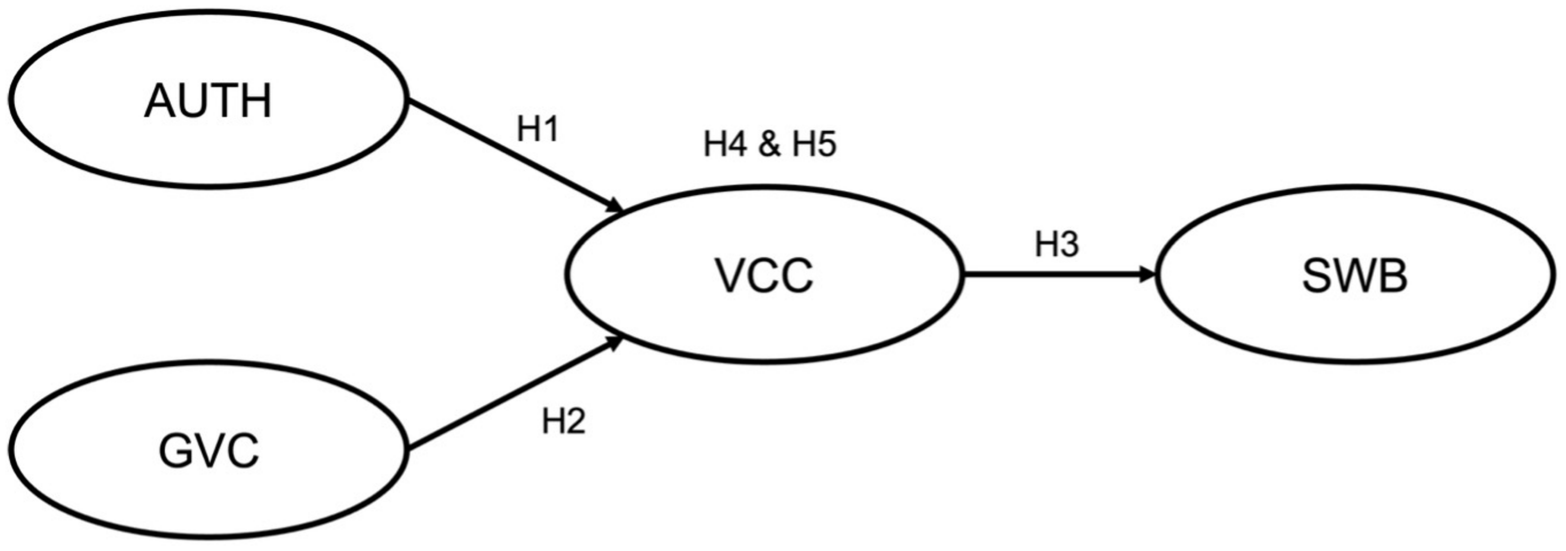
Hypothesized model of residents’ value creation and wellbeing in sport events. AUTH, perceived authenticity of event green initiatives; GVC, Resident-event green value congruence; VCC, Green value co-creation intention; SWB, Subjective wellbeing. H4 and H5 represent mediation hypotheses.

To address the research purpose, a conceptual framework was established based on SDL (see Figure 1) to examine the association between green initiatives and residents’ SWB during large-scale sport events. Specifically, drawing on the value creation tenets of SDL, this study examines how residents’ perceived authenticity of event green initiatives (AUTH) and resident-event green value congruence (GVC) relates to SWB, with green value co-creation intention (VCC) serving as a mediation.

### 3.1 The relationship between AUTH and VCC

Authenticity functions as a critical catalyst for VCC by fostering trust, facilitating reciprocal resource integration, and aligning the goals of multiple actors (13, 25). It fundamentally shapes how experiential value is generated, a dynamic that is particularly salient in the context of sport event (15, 33–35). For instance, in charity sport events, the authentic alignment of sponsors with a cause motivates residents to contribute operant resources, such as personal narratives and social networks, and operand resources, co-creating outcomes such as cause awareness and community cohesion (15).

Extending this logic to sport events’ green initiatives, we posit that residents’ perceptions of authenticity, specifically their belief that green measures are sincere and not merely performative, are positively associated with their engagement in environmental VCC (18, 36). When residents perceive these initiatives as authentic, which is evidenced by their transparent implementation, alignment with the community sustainability agenda, and non-commercial motives, they may report a greater willingness to take supportive actions. These actions included sharing eco-friendly tips, educating peers, and proactively using reusable items. This aligns with SDL’s emphasis on “genuine service exchange” driving VCC. Therefore, we hypothesize as follows:

*H1*: AUTH has a positive association with VCC.

### 3.2 The relationship between GVC and VCC

GVC, as conceptualized in the current study, is defined as the perceived alignment between an individual’s personal environmental values and the environmental responsibility values advocated by sport events (37, 38). This congruence is not an objective condition, but a subjective perception, wherein residents assess whether their own concerns regarding community environmental sustainability are aligned with those of sporting events hosted in their communities. Drawing on Congruence Theory (37), when individuals with strong environmental values perceive that an entity’s commitment to sustainability fulfills their intrinsic need, they are more likely to collaborate with and contribute to that entity. Prior studies have proposed that value congruence fosters proactive behavior. For instance, environmental fit between employees and organizations boosts employees’ creative performance in the workplace (39), while consumer-sponsor environmental value fit enhances brand support (38). In large-scale sports events, residents, as key stakeholders, are more inclined to engage in VCC, such as participating in event-related environmental volunteering and providing green operation suggestions when their environmental values match the event’s eco-oriented goals. Value congruence builds trust and intrinsic motivation. Hence, we propose the following hypotheses:

*H2*: GVC between residents and sport events has a positive association with VCC.

### 3.3 The relationship between VCC and SWB

VCC processes encompass the “procedures, tasks, mechanisms, activities, and interactions that support the co-creation of value” (40). In marketing research, VCC has been consistently shown to enhance SWB through resource integration and the fulfilment of psychological needs (14, 20). Individuals derive hedonic value and positive affect from consumption experiences, and empirical evidence reveals that purchases and pleasurable consumption may positively relate to heightened happiness (16, 28). While the intensity of the positive effect matters, the frequency of such experiences is considered central to hedonic happiness and overall SWB (20). In the context of sport events, Busser and Shulga (17) further reported that “stronger resource contribution” during VCC is positively associated with SWB. Their work also indicated that direct participation may be linked with a sense of causal ownership over outcomes which was related to the link between active engagement and SWB.

Beyond hedonic value, wellbeing is also deeply rooted in self-actualization and fulfilment, which emerge from engaging in meaningful activities that promote personal growth and self-realization (19, 27, 41). This mechanism manifests itself through eco-oriented collaboration in the context of event-related environmental VCC. When residents contribute environmental knowledge and volunteer time for waste sorting and use recycling tools in conjunction with organizers’ eco-infrastructure, they integrate resources to facilitate skill mastery, community green teamwork, and leadership in sustainability initiatives. These activities are perceived as contributing to meaningful life goals, thereby exerting a positive influence on SWB (19, 27, 41). Therefore, we hypothesize as follows:

*H3*: VCC has a positive association with SWB.

### 3.4 Mediating effect of VCC

According to Social Exchange Theory, previous research has proposed that individuals who perceive greater benefits, either tangible or intangible, are more likely to engage in VCC, which contributes to elevated SWB (19, 21, 42). However, these studies have primarily focused on direct associations between the proposed constructs. For instance, Chen et al. (19) demonstrated that residents’ perceptions of economic and sociocultural benefits from event hosting positively predicted their participation in resident-tourist VCC, which subsequently enhanced SWB. Similarly, Armbrecht et al. (42) further reported that residents’ perceived benefits, such as skill development and social identity, drive VCC engagement, with VCC acting as a key mediator between the perceived benefits and sustained SWB. Given the hypothesized relationship between residents’ AUTH and GVC and VCC, as well as the association between VCC and SWB, we postulated that residents’ VCC served as a mediator linking AUTH and GVC to SWB. These hypothesized mediations were consistent with the theoretical framework established in the literature (30, 31, 42). Hence, we hypothesize as follows:

*H4*: VCC mediates the relationship between AUTH and SWB.

*H5*: VCC mediates the relationship between GVC and SWB.

## 4 Methods

### 4.1 Research setting

This study evaluated the hypothesized model within the context of 2023HAG, a major multi-sport event held from September 23 to October 8 in Hangzhou, China. Comparable in scale to the Olympic Games but with participation confined to Asian nations, the 2023HAG distinguished itself through an exceptional commitment to environmental sustainability (43). As the first Asian game to fully incorporate the United Nations Sustainable Development Goals into its planning framework, the 2023HAG set a benchmark for sustainable large-scale sports events. Its legacy includes a comprehensive environmental blueprint that continues to influence the city’s ecological landscape and reshape residents’ daily practices (44).

The green initiatives of the 2023HAG actively fostered resident engagement by creating accessible avenues for resource integration. A notable example was the “low-carbon account” (44), an interactive digital platform that allowed residents to earn redeemable points through everyday sustainable actions such as waste sorting, plastic-free shopping, and green cycling. These points can be exchanged for eco-friendly rewards, including items made from recycled materials or official merchandise (43, 44). Moreover, the widely participated “Kilogram per Person” carbon-neutral campaign attracted tens of millions of residents, effectively transforming green practices into prevailing community norms (44). Through these lived experiences, rather than through abstract messaging, residents were able to assess the authenticity of green initiatives directly. This makes 2023HAG an ideal and robust setting for investigating how such perceptions influence residents’ SWB.

### 4.2 Measures

All constructs were measured using a seven-point Likert scale ranging from 1 (“strongly disagree”) to 7 (“strongly agree”). The measurement items were adapted from scales established in previous studies and validated in the present research context. GVC was measured using a three-item scale developed by Cable and DeRue (45) to assess the extent to which residents perceived alignment between the 2023 HAG green initiatives and their community sustainability agenda. The AUTH was assessed using an adapted five-item scale (46), which captures residents’ perceptions of the genuineness of the event’s green initiatives. VCC was evaluated using three items developed by Busser and Shulga (17) to measuring residents’ active contribution to environmental value co-creation processes. To assess SWB as an outcome, a six-item measure was adapted from Diener et al. (1), focusing on residents’ self-reported evaluations of life satisfaction.

As the original scales were developed in English, and the questionnaire was administered to Chinese respondents, a rigorous back-translation procedure following Doherty et al. (47) was utilized to ensure conceptual and linguistic equivalence between the English and Chinese versions of the questionnaire.

### 4.3 Data collection

Data were collected through a cross-sectional online survey administered through the Wenjuanxing platform to Hangzhou residents between September 23 and October 13, 2023. The written consent form served as the survey cover page which outlined the research purpose and protocols. Respondents’ decision to proceed with survey completion was deemed implicit assent to participate in the study.

Using G*Power (48), a minimum sample size of 160 was determined based on the number of measured constructs, to ensure adequate statistical power for model identification. A convenience sampling approach was applied, stratified across Hangzhou’s 13 administrative districts to ensure geographic representativeness. Eligible respondents were required to be at least 18 years old, residing in Hangzhou during the 2023 HAG, and aware of the 2023HAG green initiatives.

A total of 823 valid responses were obtained, yielding a response rate of 82.5%. The sample comprised 52% female and 48% male respondents, with an average age of 34.7 years (S.D. = 9.2). Regarding educational attainment, 68% had a bachelor’s degree or higher. With regard to occupational distribution, 45% were white-collar employees, 28% were blue-collar workers, and 27% were from other sectors. Regarding monthly household income, 22% earned less than USD 700, 49% earned between USD 701 and USD 1,500, and 29% earned more than USD 1,501.

### 4.4 Data analysis

The hypothesized model was tested using partial least squares structural equation modeling (PLS-SEM) in SmartPLS 4.1.1. PLS-SEM is a causal-predictive method well suited for exploratory research and theory development (49, 50), particularly in social science contexts where the goal is to provide causal explanations while balancing both explanatory and predictive aims.

The analysis follows the two-stage procedure recommended by Hair et al. (49). First, the measurement model was evaluated using confirmatory composite analysis. Given that PLS-SEM estimates model parameters based on partial information, conventional goodness-of-fit indices commonly used in covariance-based structural equation modeling are not applicable (50, 51). Instead, the measurement model was assessed by using multiple criteria. Internal consistency was evaluated by examining indicator loadings and composite reliability (CR), and convergent validity was evaluated using the average variance extracted (AVE) of each construct (51). Discriminant validity was tested using two established methods: the Fornell-Larcker criterion (52), which compares the square root of the AVE with inter-construct correlations, and the heterotrait-monotrait ratio (HTMT) of correlations (53).

In the second stage, the structural model was examined to evaluate its predictive power and substantive relationships. This involved testing for potential multicollinearity among endogenous constructs, examining the statistical significance and magnitude of path coefficients, and evaluating the model’s explanatory power through the coefficient of determination (*R*^2^), Additionally, the model’s predictive relevance was assessed using blindfolding-based cross-validated redundancy measure (*Q*^2^), following established guidelines for PLS-SEM validation (50, 51).

## 5 Results

### 5.1 Testing of normality and multicollinearity

Prior to assessing the measurement and structural models, the data were screened for normality and multicollinearity. Normality was examined using the Skewness-Kurtosis test (51, 54). Table 1 shows that values ranged from −1.23 to 0.67 for skewness and from −0.78 to 5.45 for kurtosis, indicating no substantial deviation from data normality. The Variance Inflation Factor (VIF) test was then performed for multicollinearity among all factors for analysis. The VIF values of AUTH and GVC are 1.18 and 1.20, suggesting that multicollinearity was not a concern in this study (51, 54).

**Table 1:** Skewness, kurtosis and VIF values for latent constructs.

Latent construct	Skewness	Kurtosis	VIF
AUTH	−1.23	4.12	1.18
GVC	−0.86	5.45	1.20
VCC	0.24	−0.78	—
SWB	0.67	2.31	—

VIF was only calculated for exogenous constructs (AUTH, GVC); thresholds: |Skewness| < 2, |Kurtosis| < 7, VIF < 3.

### 5.2 Testing of common method variance

As the data were collected through self-reported measures from Hangzhou residents, potential common method variance (CMV) was addressed both procedurally and statistically. During the survey design, several strategies recommended by Podsakoff et al. (55) were implemented, including the use of validated scales, separation of independent (e.g., AUTH, GVC) and dependent (e.g., SWB) variables, and item randomization, to mitigate social desirability bias (55). After the data collection, Harman’s single-factor test was performed. The results showed that the first unrotated factor only accounted for 30.3% of the variance, which was far below the 40% threshold, indicating a severe CMV. Thus, CMV was not considered a threat to the validity of the findings.

### 5.3 Testing of the measurement model

Assessment of the measurement model involved evaluating four reflective constructs: AUTH, GVC, VCC, and SWB. As summarized in Table 2, all factor loadings ranged from 0.71 to 0.88 and exceeded the recommended cutoff value of 0.70 (51). The CR values varied from 0.83 to 0.91, surpassing the acceptable threshold of 0.70 (51, 52). These indicators suggested the reliability of the measurement model. Convergent validity was supported by the AVE values of all constructs surpassing the 0.50 threshold (52). With respect to discriminant validity, Table 2 reveals that the square roots of AVE for all constructs exceeded their correlations with the other constructs. A more robust measure of HTMT further affirmed discriminant validity by showing HTMT values below the recommended value of 0.85 (see Table 3) (53).

**Table 2:** Standardized factor loadings, construct reliability, and average variance extracted for all scales.

Constructs	Indicator loadings	CR	AVE
Perceived authenticity of green initiatives (AUTH)		0.89	0.57
The Asian Games’ environmental performances are genuine.	0.81		
The environmental performances preserve what the Asian Games means to me.	0.71		
The Asian Games are true to itself with its environmental performances.	0.74		
The Asian Games stand up for what it believes in.	0.77		
The Asian Games are environment friendly.	0.73		
Green value congruence (GVC)		0.88	0.58
My personal values match 2023HAG’s values and culture.	0.80		
2023HAG’s values provide a good fit with the things that I value.	0.77		
The things that I value in life are similar to those 2023HAG values.	0.75		
Value co-creation (VCC)		0.86	0.62
I contribute my experience to pro-environmental performances.	0.87		
I invest my resources in pro-environmental performances.	0.78		
I made a personal investment in this.	0.72		
Subjective wellbeing (SWB)		0.91	0.63
I have felt cheerful and in good spirits.	0.79		
I have felt calm and relaxed.	0.77		
I have felt active and vigorous.	0.78		
I woke up feeling fresh and rested.	0.77		
My daily life has been filled with things that interest me.	0.83		
I am optimistic about my future.	0.84		

*N* = 823. CR, Construct Reliability; AVE, Average Variance Extracted.

**Table 3:** Descriptive statistics, reliability estimates and correlations for the study variables.

	AUTH	GVC	VCC	SWB	*M*	S.D.
AUTH	**0.75**	0.57	0.56	0.42	6.06	1.01
GVC	0.44	**0.76**	0.63	0.59	5.99	1.04
VCC	0.30	0.46	**0.79**	0.59	5.73	1.32
SWB	0.42	0.43	0.41	**0.79**	5.58	1.21

*N* = 823. AUTH: perceived authenticity of event green initiatives; GVC, Resident-event green value congruence; VCC, Green value co-creation intention; SWB, Subjective wellbeing. Diagonal elements (in bold) represent the square root of the AVE. Below-diagonal elements are construct intercorrelations. The diagonal elements above are HTMT ratios. M, mean; S.D., standard deviation.

### 5.4 Testing of the structural model

**Figure 2:**
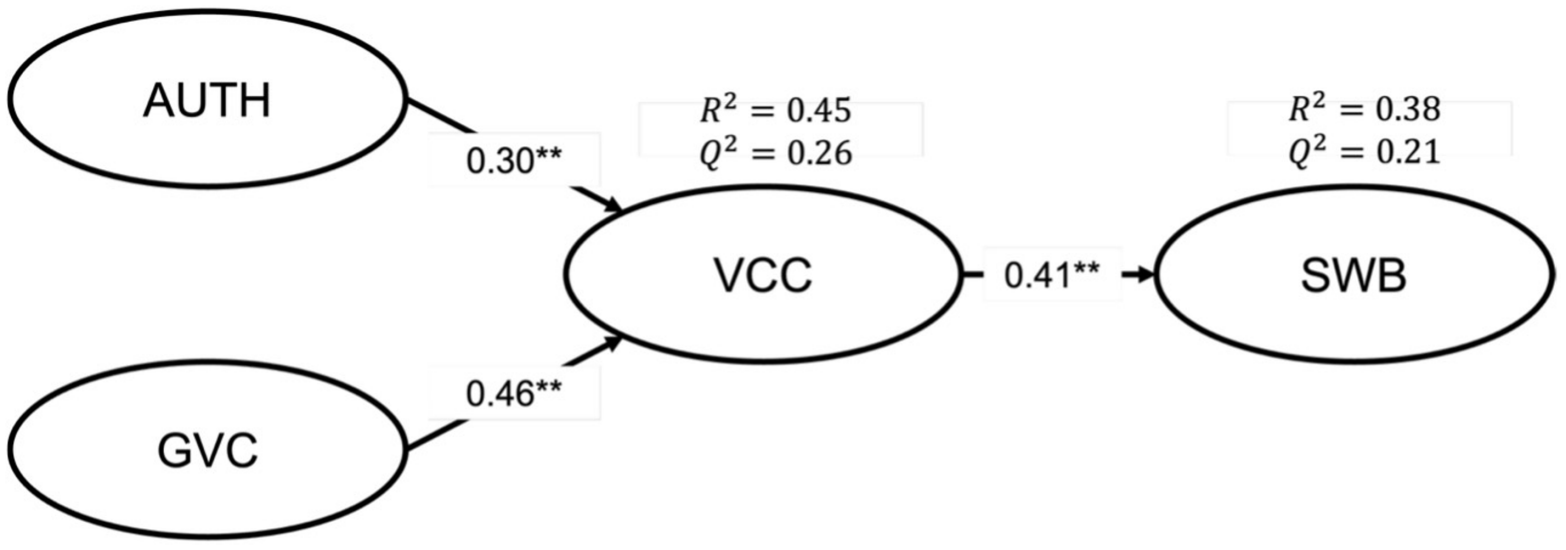
Results of direct path coefficients and predictive capability. AUTH, perceived authenticity of event green initiatives; GVC, Resident-event green value congruence; VCC, Green value co-creation intention; SWB, Subjective wellbeing. *R*^2^, coefficient of determination; *Q*^2^, cross-validated redundancy measure. *p < 0.05, **p < 0.01.

Bootstrapping procedure with 5,000 samples were employed to test the hypothesized relationships in the structural model. The results in Figure 2 shows that AUTH was positively associated with VCC (*β* = 0.30, *t* = 3.96, *p* < 0.01), and GVC was significantly related to VCC (*β* = 0.46, *t* = 5.33, *p* < 0.01). Thus, H1 and H2 are supported. Furthermore, VCC exhibited a positive association with SWB (*β* = 0.41, *t* = 5.43, *p* < 0.01), supporting H3.

With respect to the explanatory power of the model, PLS_predict_ was used to calculate the *R*^2^ and *Q*^2^ values for VCC and SWB. Figure 2 indicates that the VCC (*R*^2^ = 0.45, *Q*^2^ = 0.26) and SWB (*R*^2^ = 0.38, *Q*^2^ = 0.21) had adequate predictive capability (50, 51).

### 5.5 Testing of the mediating effect

This study assumed that the associations of AUTH and GVC with residents’ SWB were mediated by VCC. Therefore, we assessed the indirect relationships of each path. Table 4 shows that the indirect association of VCC was significant within the relationship between AUTH and SWB, as its 95% confidence interval (CI) excluded zero [*β* = 0.08, *t* = 4.50, *p* < 0.01, 95% CI = (0.05,0.12)]. Similarly, the indirect association of VCC was significant in the link between GVC and SWB [*β* = 0.11, *t* = 5.37, *p* < 0.01, 95% CI = (0.07, 0.14)]. Hence, H4 and H5 are supported. The VCC fully mediated the relationship between AUTH and SWB. Additionally, a significant direct association was found between GVC and SWB (*β* = 0.21, *t* = 3.01, *p* < 0.01), suggesting that the GVC–SWB association was partially mediated by VCC.

**Table 4:** Summary of standardized path coefficients of the hypothesized paths.

	Paths	*β*	*SE*	Bootstrapping (95% CI)		Results
				2.5% Lower	2.5% Upper	
Direct effect						
H1	AUTH → VCC	0.30**	0.06	0.16	0.45	Support
	AUTH → SWB	0.06	0.08	−0.06	0.16	
H2	GVC → VCC	0.46**	0.08	0.29	0.62	Support
	GVC → SWB	0.23*	0.09	0.09	0.38	
H3	VCC → SWB	0.41**	0.08	0.26	0.56	Support
Indirect effect						
H4	AUTH → VCC → SWB	0.13*	0.04	0.05	0.22	Support
H5	GVC → VCC → SWB	0.19**	0.05	0.11	0.29	Support

*N* = 823. AUTH: perceived authenticity of event green initiatives; GVC, Resident-event green value congruence; VCC, Green value co-creation intention; SWB, Subjective wellbeing. β, standardized coefficient; SE, Standard error; CI, Confidence interval. *p < 0.05, **p < 0.01.

## 6 Discussion

This study examined how residents’ perceptions of green initiatives relate to their SWB within the context of 2023HAG, a large-scale sports event. Building on SDL, this study developed and tested a conceptual framework containing the constructs of AUTH, GVC, VCC, and SWB, which was then tested using PLS-SEM. The findings provide robust support for the hypothesized relationships and yield several theoretical implications that advance current understanding in event management and public health.

The positive association between AUTH and VCC (H1) verifies the role of authenticity as a vital antecedent of VCC under the SDL framework. Prior research has consistently shown that authenticity builds trust and lowers barriers for resource integration and collaborative actions (15, 33, 56). Our finding extends this logic to the environmental domain by showing that residents who perceive green initiatives as genuine are more willing to form intention to contribute operant resources such as eco-friendly knowledge and practical suggestions. This responds to widespread skepticism about “greenwashing” among large-scale event (32, 33) and identifies constructive authenticity as a powerful antecedent of green VCC. In doing so, we enrich SDL scholarship by showing that genuine service exchange can be activated through an event’s symbolic commitment to sustainability.

The significant relationship between GVC and VCC (H2) indicates that when an event’s sustainability mission mirrors community priorities, residents are far more likely to engage in value co-creation behaviors. While previous research shown that value congruence can effectively arouse stakeholders’ co-creation intention and further drive positive emotional experiences (38, 39, 57), its role for involuntary, passive resident stakeholders in large-scale events has been underexplored. We identify GVC as a mechanism that transforms passive residents into active resource integrators, aligning with SDL’s core premise that value propositions must resonate with actors’ values to elicit reciprocal exchange (13, 25).

The positive connection between VCC and SWB (H3) supports that residents’ intention to engage in eco-oriented collaboration is closely associated with their enhanced SWB (17). The willingness to participate in green VCC brings positive experiences via anticipated social interaction and a prospective sense of collective achievement. Our finding is consistent with extant research stating that frequent positive psychological experiences improve individual SWB (17, 20). This substantiates SDL’s claim that value is phenomenologically determined by beneficiaries, and that active resource integration directly shapes wellbeing outcomes.

Our mediation analyses reveal distinct mechanistic differences. The full mediation of VCC between AUTH and SWB (H4) suggests that authenticity alone is insufficient to enhance SWB. Instead, it must translate into active VCC intention to exert influence. This refines authenticity scholarship by positioning authenticity as a distal enabling condition, with resource integration serving as the proximate driver of psychological benefits, which directly echoes SDL’s tenet that value cannot be unilaterally delivered (13, 14). In contrast, the partial mediation between GVC and SWB (H5) reveals a more complex dynamic. While VCC remains a key mechanism, value congruence itself enhances wellbeing by reducing cognitive dissonance and fostering collective identity affirmation, consistent with Self-Congruity Theory (37, 38). This extends SDL by suggesting that deeply congruent value propositions can generate immediate psychological benefits even before formal resource integration occurs.

This study measures residents’ value co-creation intention rather than their actual behaviors. From a theoretical perspective rooted in SDL, intention is not an ultimate outcome but a pivotal transitional stage (13, 17, 18). Residents who hold strong green co-creation intention will gradually engage in practical pro-environmental and health behaviors (17). The positive psychological state and enhanced subjective wellbeing brought by VCC can further reinforce residents’ behavioral persistence. In the context of large-scale sports events, the collective atmosphere and community norms formed by green initiatives can reduce the barriers between intention and action, prompting residents to turn their willingness to collaborate into daily environmental and health practices. This intention-behavior linkage is an indispensable part of the SDL-based value co-creation ecosystem.

### 6.1 Theoretical implications

This study makes several key theoretical contributions to event and public health literature. First, it extends the application of SDL by demonstrating its relevance to residents of host communities who do not actively participate in large-scale sporting events. While prior research has documented improvements in residents’ SWB following sporting events (2, 8), most studies have primarily focused on actual co-creation behaviors of active participants, such as players and spectators (7, 16). Context has been recognized as a crucial factor in understanding the value-creation process in sport settings (15, 16), and this research reveals meaningful distinctions between the experiences of passive residents and those of active participants identified in earlier work. Building on Busser and Shulga’s (17) initial work, this study developed an SDL model and empirically examined how residents’ pro-environmental VCC intention influences their SWB. The results align with the marketing literature (58), indicating that the greater the resources an individual integrates into the VCC process, the higher the perceived levels of value and wellbeing they will perceive. Importantly, this process is independent of an individual’s role as an active or a passive participant. Therefore, our model contributes to the literature by highlighting the benefits, specifically in terms of SWB, of residents passively involved in large-scale sports events.

The current study furthers the power of the Social Leverage Model in explaining how hosting sport events generates social benefits for host communities. In addition to the celebratory atmosphere and social camaraderie (11), Inoue and Havard (22) and Wu et al. (59) added events’ social responsibility as a third dimension of social leverage. Previous studies primarily focused on celebratory atmosphere and social camaraderie as driving factors for individuals’ happiness and SWB (2). In these studies, SWB was often explained by a “feel-good effect” arising from the celebratory atmosphere, social interaction, and leisure engagement. In comparison, the present study considered event green initiatives as the entry point, and integrated SDL to explore the social leverage effect generated by residents’ value co-creation intention. According to Asan et al. (21), residents are more likely to generate collaborative intention when they recognize the authenticity and community compatibility of event green projects. Such positive co-creation intention further satisfies residents’ intrinsic need for community contribution and increased individual SWB. This demonstrates that sports events can enhance SWB by framing residents as active “eco-resource integrators,” thereby providing a more holistic theoretical account of how events contribute to residents’ wellbeing.

This study provides empirical support for a significant positive association between VCC and values on beneficiaries’ wellbeing. It reinforces the pivotal role of authenticity and meaningful engagement in linking sport events to community welfare. Event and tourism research has consistently shown that perceived authenticity is essential in shaping residents’ SWB in communities hosting sporting events (21, 31, 60). Our findings align with the literature, demonstrating that sporting events that authentically reflect the values and identities of host communities are far more likely to garner resident engagement and support (21, 31). Specifically, when residents invest time and effort in events through a VCC, this engagement leads to enhanced perceptions of value and subsequent improvements in SWB. The value derived from the VCC is phenomenologically more enduring, relational, and beneficiary-specific than the benefit-over-cost framework of value. Driven by meaningful personal resource contributions, individuals not only assign higher instrumental value to their engagement, but also experience greater intrinsic value, an outcome directly indicative of elevated SWB. Our results further suggest that beneficiaries prioritize personal resources only when they serve instrumental purposes, functioning as a means of achieving outcomes with greater intrinsic worth.

### 6.2 Managerial implications

This study offers actionable managerial insights for large-scale sports event organizers and policymakers seeking to leverage environmental initiatives to boost residents’ engagement and enhance welfare. First, event organizers should prioritize authenticity in green initiatives to build trust and potentially motivate resident involvement in value co-creation activities. Authenticity demands moving beyond performative greenwashing to implement transparent-purpose-driven eco-measures. This requires open communication about environmental objectives, such as carbon neutrality targets and waste diversion rates, with progress shared via accessible channels including local newsletters, social media platforms, and community meetings. Such transparency ensures that residents understand the initiative’s genuine intent and real-world impact.

Second, residents should be involved in the co-design of green projects through focus groups or public surveys to integrate local environmental knowledge and priorities. For instance, neighborhoods with high landfill pressure might prioritize waste reduction initiatives, whereas areas lacking green space could focus on tree-planting efforts. Delivering tangible, measurable outcomes such as visible improvements in local air quality or documented reductions in event-related waste reinforces that initiatives are meaningful contributions to sustainability, rather than superficial marketing tactics.

Finally, it is critical to lower residents’ participation barriers and diversify engagement channels to facilitate their engagement in VCC. Not all residents can commit to intensive volunteer roles, such as full-day waste sorting; therefore, policymakers and event organizers should offer flexible, low-effort opportunities. These include using event-provided recycling kits at home, sharing eco-friendly tips on social media, or attending short educational workshops. Providing practical resources such as free recycling bags, compost bins, or digital tools to track personal environmental contributions further facilitates participation. Publicly recognizing resident efforts through community impact metrics featured resident stories in event communications or appreciation events that reinforced the psychological rewards of involvement, strengthening residents’ sense of belonging and self-worth.

### 6.3 Limitations and future research directions

This study had some limitations that warrant attention. First, the cross-sectional survey design captures only a snapshot of residents’ perceptions and behavioral intentions during the Hangzhou Asian Games, precluding definitive causal inferences between residents’ perceptions of green initiative authenticity and fit, value co-creation intention, and SWB. It is impossible to verify the long-term public health impacts of event green initiatives. The widespread excitement of hosting a large-scale sport event may have artificially inflated short-term SWB. Therefore, future research should adopt a longitudinal design to collect data at multiple time points, including the pre-, during-, and post-event phases, to examine temporal dynamics and validate causal pathways.

Second, the use of convenience sampling via an online panel platform may limit the generalizability of our findings. Specifically, 68% of respondents held a bachelor’s degree which is above the general adult population in Hangzhou. This educational overrepresentation may lead to an overestimation of the strength of associations between green perceptions and SWB, as education level is linked to greater environmental awareness. To address the limitation, future studies should employ more robust sampling design to ensure balanced representation across key demographics including age, education, income, and geographic location within the host city, thereby enhancing the external validity.

Third, the current study focused on Hangzhou Asian Games as a specific context, which limits the generalizability of the findings. Unique contextual factors, such as local policy support for sustainability, cultural attitudes toward environmental initiatives, and the event’s scale of green investment, may influence the results. Future research should replicate the model across different large-scale sports events, such as the Olympics, the FIFA World Cup, or other international tournaments, and diverse cultural contexts to test its robustness.

Finally, this study is limited to measuring value co-creation intention rather than actual health and environmental behaviors, leaving the intention-behavior transformation untested. Future studies can integrate actual behaviors into the SDL framework, empirically examine how co-creation intention translates into real actions, and analyze key factors influencing this transition process in mega-event settings.

## Data Availability

The raw data supporting the conclusions of this article will be made available by the authors, without undue reservation.
